# Biomaterials in periapical regeneration after microsurgical endodontics: A narrative review

**DOI:** 10.4317/jced.58651

**Published:** 2021-09-01

**Authors:** Paloma Montero-Miralles, Rafael Ibáñez-Barranco, Daniel Cabanillas-Balsera, Victoria Areal-Quecuty, Benito Sánchez-Domínguez, Jenifer Martín-González, Juan J. Segura-Egea, María C. Jiménez-Sánchez

**Affiliations:** 1 DDS, MSc, PhD, Professor of Master in Clinical Endodontics, University of Sevilla, C/ Avicena s/n, 41009-Sevilla, Spain; 2 DDS, MSc, Doctoral fellow, Department of Stomatology, Section of Endodontics, School of Dentistry, University of Sevilla, C/ Avicena s/n, 41009-Sevilla, Spain; 3 DDS, MSc, PhD Associate Professor, Department of Stomatology, Section of Endodontics, University of Sevilla, C/ Avicena s/n, 41009-Sevilla, Spain; 4 MD, DDS, PhD, Professor, Department of Stomatology, Section of Endodontics, School of Dentistry, University of Sevilla, C/ Avicena s/n, 41009-Sevilla, Spain; 5 DDS, MSc, PhD, Materials Science Institute of Sevilla (ICMS), Joint CSIC-University of Sevilla Center, 41092 Sevilla, Spain

## Abstract

**Background:**

The objective of this narrative review was to analyze the available scientific evidence regarding the application of biomaterials in endodontic microsurgery and its influence in post-surgical tissue repair.

**Material and Methods:**

The review question was Do biomaterials used in endodontic microsurgery influence post-surgical tissue repair and regeneration? Systematic MEDLINE/PubMed review was used to evaluate and present the results.

**Results:**

The search yielded 131 references, 82 of which were selected for full text review after reading the abstracts. After a manual search in the references of the articles selected, 52 references were eliminated. Finally, 30 articles were selected.

**Conclusions:**

Bone grafts, membranes and bioceramics, especially MTA, are biomaterials with the ability to stimulate periapical tissue regeneration. This is one of many reason why bioceramics are the best choice as retrograde sealing materials. However, microsurgically treated periapical lesions can heal completely without the need to use bone grafts or membranes. Those techniques are indicated in endodontic microsurgery when additional stimulation of tissue regeneration is required, or when bone collapse needs to be prevented.

** Key words:**Bioactive endodontic cements, endodontic surgery, periapical repair.

## Introduction

When conventional endodontic treatment does not achieve its goals, the periapical lesion persists (persistent chronic apical periodontitis), and endodontic surgery may be indicated at that moment. A distinction must be made between traditional endodontic surgery and endodontic microsurgery, in which the dental operating microscope (DOM) is used (1). Both treatments´ aim is the retrograde retreatment of the root canal system, but endodontic microsurgery combines magnification and illumination, provided by the DOM, with the appropriate use of micro-instruments to perform a microsurgical approach to the periapical area.

Endodontic microsurgery is indicated when orthograde root canal treatment or retreatment fails to achieve its goal, of preventing or curing apical periodontitis, and the periapical lesion is not repaired. In the bibliographic review by Setzer *et al*. (2) comparing the success rates of traditional periapical surgery and endodontic microsurgery, it is concluded that the success rate is 59% for traditional surgeries and 94% for endodontic microsurgeries. Increasing the success rate of endodontic microsurgery not only depends on the use of DOM; the use of new biomaterials for retro-filling, such as mineral trioxide aggregate (MTA) (3) and other bioceramics cements, as well as the biomaterials used for bone regeneration (4) has also played an important role. Biomaterials are biocompatible and pharmacologically inert materials that are designed to be in contact and interact with biological systems, with the purpose of evaluating, treating, increasing, repairing or replacing some tissue function of the human body (5).

When the size of the periapical lesion is very large, it is also possible to perform guided bone regeneration techniques (ROG) with biomaterials, such as bone grafts and membranes, to avoid a non-osteogenic tissue regeneration pattern, very different from the original structures in the area of injury (6).

Newly formed bone filling of the surgical wound after endodontic surgery is an essential step in periapical repair, which is why different strategies have been proposed to stimulate apposition and new bone formation. The autologous bone graft, taken from the same patient, is the reference biomaterial to achieve bone repair (5), since it has the properties of osteogenicity, osteoinductivity and osteoconductivity, in addition to being non-immunogenic. On the other hand, the use of homologous bone (taken from another individual of the same specie) or heterologous (taken from another species) has the disadvantage of immune rejection reactions and the possibility of infectious disease transmission. This has led to the development of synthetic alloplastic biomaterials that contribute to bone repair. The first generation of synthetic biomaterials for bone regeneration included some metals, synthetic polymers and ceramic materials. A second generation followed, including synthetic and natural biodegradable polymers, synthetic or naturally occurring calcium phosphates (bovine bone), natural or synthetic calcium carbonates, calcium sulfates and bioactive crystals. The third generation of biomaterials has been designed to incorporate signaling molecules that improve stem cell survival and direct their differentiation towards a specific cell line (5). This group includes the use of soluble factors (growth factors, cytokines), the use of insoluble factors (extracellular matrix molecules) or the use of external incentives (mechanical load, compressive stress, bending stress, conductive polymers).

The objective of this narrative review was to analyze the scientific evidence regarding the effect of biomaterials applied in endodontic microsurgery on post-surgical tissue repair and regeneration.

## Material and Methods

A bibliographic search was carried out using the database MEDLINE-PubMed using the following keywords: (biomaterial OR membrane OR bioceramic OR biocompatible material OR bioactive) AND (healing OR repair OR regeneration) AND (endodontic OR periapical OR apical OR periradicular OR rootend) AND (microsurgery OR microscope OR microscopic).

The inclusion criteria established were studies performed in humans or animals until December 2020, if possible with at least one year follow-up. Case reports and studies based on surveys or expert opinions were excluded. No language restriction was applied. When there was no initial agreement among the reviewers, consensus was reached through dialogue.

A hand-search was also carried out in main endodontic journals (International Endodontic Journal, Journal of Endodontic, and Australian Endodontic Journal) and in the references of significant papers and reviews. The last search was made on March 2021.

Electronic and manual searches provided the titles and abstracts of articles related to the aims of the studies, which were categorized by two independent researchers (D.C-B. and J.J.S-E.) according to the inclusion and exclusion criteria. Articles selected were full-text reviewed by five investigators (P.M.-M., R.I.-B.,D.C-B., J.M-G, and J.J.S-E).

## Results

The search yielded 131 references, 82 of which were selected for full text review after reading the abstracts. After a manual search in the references of the articles selected, 52 references were eliminated. Finally, 30 articles were selected. Of all the articles reviewed, 4 of them recommend the use of a bioceramic material as the ideal retro-filling material (7,9,17,18). Four studies analyzed clinical results in cases of endodontic microsurgery versus conventional periapical surgery (6,7,28,29). On the other hand, 4 studies analyzed periapical microsurgery results using biomaterials as membranes or bone grafts (4,7,18,28). Finally, 7 articles reported clinical studies in humans on periapical repair after endodontic microsurgery, with controls of at least one year (19-25).

## Discussion

Retro-filling is an essential part of periapical surgery. The ideal retro-filling material should seal the root canal system, preventing the extrusion of bacteria and their by-products into the surrounding periradicular tissues. Their characteristics must include minimal cytotoxicity, bactericidal or bacteriostatic properties, good sealing capacity of the retro-preparation, good dimensional stability and being non-resorbable, biocompatible and bioactive to promote the formation of cement and bone.

**Figure 1:**
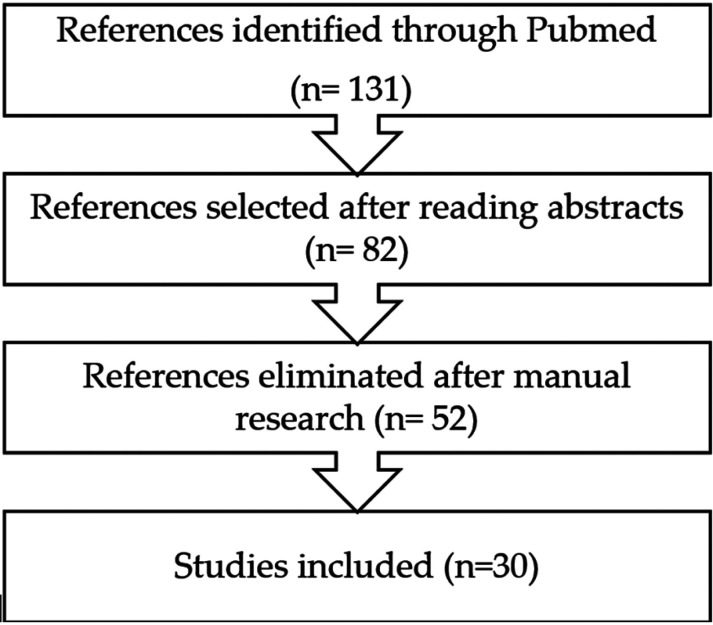
Flow diagram showing the process by which the studies were selected.

Throughout history, numerous materials have been used for retro-filling, with all of them achieving repair of the periapical lesion (7) (Fig. 1). Silver amalgam has been one of the most used retro-filling materials, but its use has been stopped due to mercury toxicity, corrosion, expansion, electrolysis, tissue tattooing, not preventing microfiltration and not allowing the regeneration of dentoalveolar structures (3,8,9). In 1978, Oynick suggested Super-EBA as a retro-sealing material. It is a reinforced zinc oxide-eugenol cement, and it has multiple advantages over silver amalgam, such as sealability, periapical tissue reaction, and regeneration of periapical tissues (10).

In the 90s, with the introduction of the first bioceramic material, MTA (Mineral Trioxide Aggregate) (3), a new era began for endodontics, and especially for endodontic surgery. Bioceramics are ceramic materials with excellent biocompatibility, good sealability, and not adversely affected by contamination with blood and periradicular fluids (8). They also increase cell proliferation and promote mineralization (3,9). In histological studies it has been shown that bioceramics have a strong potential for cement regeneration on the retro-filling material (8,11,12).

Recently, new bioceramic cements for back-filling have reached the market. Endoquence is a hydrophilic, radiopaque, aluminum-free bioceramic. Several *in vitro* and *in vivo* studies have shown that it is a non-toxic, biocompatible material, it does not undergo shrinkage and it is sTable over time, providing good sealing capacity. Furthermore, it shows antibacterial activity against E. faecalis, possibly due to its alkaline pH (13).

The first studies on the possible regenerative potential of bioceramic materials were performed in cell cultures, mainly in fibroblasts and in pulp, periodontal and bone marrow stem cells. Several studies have confirmed that MTA stimulates the differentiation of periodontal and gingival fibroblasts in cultures, increasing the levels of alkaline phosphatases (14), as well as cell adhesion and proliferation. Both the MTA and the Endosequence Root Repair Material (ERRM) have been shown to be biocompatible, have low toxicity, and have the ability to stimulate adhesion, proliferation and cell survival of periodontal, pulp and bone marrow stem cells (15) as well as showing positive effects on osteoblastic differentiation (16).

In the last decade, there have been many studies researching the use of bioceramics as retro-filling material in conventional endodontic surgery. MTA has usually shown better periapical reparative responses than amalgam, IRM, or Super-EBA cement. Torabinejad *et al*. (9) found a better reparative response after conventional apical surgery in dogs to MTA (with cementum formation and less inflammation) than to amalgam. Economides *et al*. (17) found hard tissue formation around the MTA, but not around IRM, after conventional periapical surgery in dogs.

Similarly, studies carried out in dogs, with conventional periapical surgery, have shown that cement forms on the gray MTA used in retro-filling, while it does not form on the zinc oxide-eugenol cement (18). In monkeys, greater cement formation and less inflammation was observed 5 months after MTA placement in the retro-filling, when compared with amalgam. In short, most studies conclude that MTA is associated with better periapical reparative responses than other bioceramic materials developed later. It has also been studied whether the use of biomaterials for bone replacement and/or membranes, in combination with bioceramics, improve periapical repair. The data from the scientific literature indicate that conventional periapical post-surgery tissue repair, using MTA as a retro-filling material, is not influenced by the use of membranes and bone graft. Regarding clinical studies in humans, this review found 7 articles in which results on periapical repair post-endodontic microsurgery are presented (Table 1).

**Table 1:**
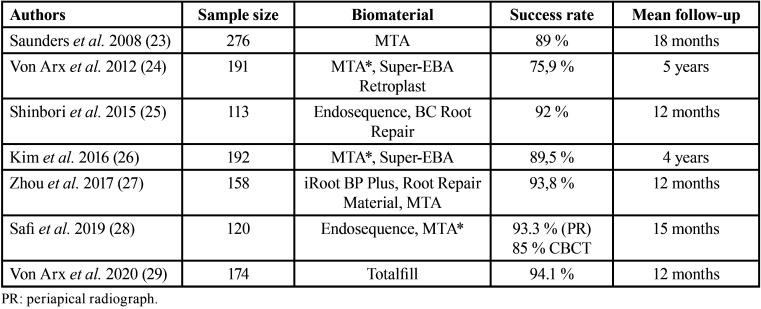
Clinical human studies where post-endodontic microsurgery periapical reparation was studied with different biomaterials.

Saunders *et al*. (19) conducted a prospective study on 276 teeth apicoectomized with apical microsurgery in which MTA was used in the retro-filling. The success rate was 89%. Von Arx *et al*. (20) evaluated patients 5 years after undergoing periapical microsurgery using three types of retro-filling materials: MTA, Super-EBA and Retroplast. The overall success rate was 75.9%. The study concludes that the healing rate was higher using MTA as retro-filling material, and when the mesio-distal interproximal bone level was 3 mm or less from the amelocemental union. Shinbori *et al*. (21) studied the clinical and radiographic outcome using EndoSequence BC Root Repair as a retro-obturation material in periapical microsurgery in 113 treated teeth evaluated 12 months after the procedure. Treatment success was found in 92% of treated teeth. Kim *et al*. (22), continuing their previous study published in 2012, reviewing 182 teeth four years after microsurgery. They found an average success rate of 89.5%, 91.6% in the MTA group and 89.9% for the Super-EBA group, without finding significant differences. In the prospective randomized controlled trial published by Zhou *et al*. (23), the results of endodontic microsurgery were clinically and radiographically evaluated using a new bioceramic material, the iRoot BP Plus Root Repair Material (BP-RRM), or MTA as retro-filling material. The success rate found for MTA and BP-RRM, 93.1% and 94.4% respectively (*p*> 0.05). In the randomized clinical trial conducted by Safi *et al*. (24), evaluating the success in healing after 15 months of periapical microsurgery in a sample of 120 teeth, comparing EndoSequence and MTA, they obtained a mean success rate of 93.3% using periapical radiography and 85% for the evaluation with CBCT. Von Arx *et al*. (25) published a study with 174 teeth that underwent endodontic microsurgery, and were filled with Totalfill, presenting a success rate of 94.1% in a one-year control.

The introduction of the operating microscope in endodontic surgery has led to a significant increase in success rates, which have gone from 60% in conventional endodontic surgery to 94% using microsurgical techniques (2) with magnification and ultrasonic tips. Histological studies carried out in animal and human models demonstrate that complete cure of large postsurgical periapical lesions can be achieved without the need to use bone grafts or guided tissue regeneration (GTR) membranes (7,18,26). GTR is a surgical therapeutic procedure that seeks to regenerate lost periodontal structures (bone, cementum and periodontal ligament). These GTR techniques have been used in conventional endodontic surgery, alone or in combination with the filling of the surgical wound using bone grafts. Several studies have found that the use of GTR techniques, alone or in combination with bone substitutes, improves neocement formation and stimulates osteoblasts (27). However, other authors conclude that although GTR combined with bone analogues increase the tissue regenerative response post-periapical surgery (4), there is insufficient evidence to demonstrate that the use of membranes alone in GTR improves bone repair after surgery for periapical lesions, with or without concomitant periodontal injury and, especially, in 4-wall lesions (4).

The first prospective clinical study introducing magnification with loupes to 4.3X in endodontic surgery, carried out by Taschieri *et al*. (28), monitored regeneration in wide apical lesions, with or without tissue regeneration techniques using resorbable collagen membrane and bovine bone. They concluded that the use of GTR in association with bovine bone in the treatment of these lesions of endodontic origin did not provide benefits in terms of regeneration. However, they recommended placing an inorganic bovine bone substitute (Bio-Oss) and a resorbable collagen membrane (BioGuide) to prevent bone collapse in through-and-through defects. These same authors (28) agree with Tsesis *et al*. (29) regarding the size of the postsurgical lesion influencing periapical healing.

The review carried out by Lin *et al*. (26) on the use of membranes, bone grafts and growth factors in conventional periapical surgery, concludes that the application of membranes for GTR or bone grafts does not ensure complete periapical regeneration, since these materials are not capable of attracting stem cells and stimulating them to differentiate into osteoblasts and cementoblasts. In fact, the use of membranes has not been shown to have a clear benefit in the regeneration of periapical tissues, except in apicomarginal bone defects caused by endoperiodontal lesions or large periapical lesions communicating with the alveolar ridge.

The use of RTG membranes in combination with platelet-rich-plasma (PRP) has also been proposed. Goyal *et al*. (30) conducted a clinical trial in patients with apicomarginal defects comparing the results obtained with collagen membranes (Healiguide®), PRP or PRP combined with collagen sponge, in the surgical treatment of chronic suppurative apical periodontitis with apicomarginal communication. The healing ratio was 83%, 89%, and 80% in the PRP, PRP with collagen sponge, and only GTR membrane groups, respectively. They conclude that PRP or PRP with collagen have similar effects to GTR with resorbable membranes in reducing the size of the periapical lesion.

Periapical regeneration is understood as a process where the affected apical tissues are replaced by a new tissue in its function and architecture, and the damaged cells are replaced by healthy cells. With regard to postsurgical regeneration with RTG in endodontic microsurgery, very few studies have been published investigating this issue.

The retrospective study by Taschieri *et al*. (6), with a 4-year follow-up, investigated the outcome of GTR in endodontic microsurgery in the treatment of through-and-through defects. The authors conclude that, in this type of defects, evaluating the results through clinical and radiographic criteria of success and failure, the use of GTR combined with microsurgery gives excellent results. On the other hand, Tsesis *et al*. (29) conducted a systematic review with meta-analysis on the influence of GTR on the outcome of conventional surgical endodontics. They concluded that there is a tendency for better results when GTR is used, with the size and type of lesion influencing the result, as well as the type of membrane used. GTR techniques favorably affect the outcome of endodontic surgery treatments in cases of large periapical lesions and through-and-through defects, because in large lesions the uptake and differentiation of progenitor stem cells into odontoblasts and cementoblasts is more complicated. However, GTR does not significantly improve results on 4-wall defects. Furthermore, the results are more favorable when a resorbable membrane is used, compared to a non-resorbable membrane or only a graft.

## Conclusions

Endodontic microsurgery represents a significant increase in the success rate when compared to conventional endodontic surgery, thanks to the use of magnification, lighting and micro-instruments that allow a more precise treatment and a minimally invasive approach. The overall success rate was 91.5% at 5 years and 93.3% at 10 years. Currently, the use of DOM is considered essential to perform this surgical treatment. Bone grafts or membranes are not needed to achieve complete healing in surgically treated periapical lesions, but they can be used to improve tissue healing, especially to prevent possible bone collapse. MTA is a biocompatible material with a cementogenic capacity, which promotes periapical healing through regeneration. More studies are needed to evaluate the new bioceramic cements with endodontic microsurgery techniques and long-term follow-up.
